# Endometriosis: disease mechanisms and health disparities 

**DOI:** 10.2471/BLT.24.292660

**Published:** 2024-11-02

**Authors:** Nilufer Rahmioglu, Krina T Zondervan

**Affiliations:** a Nuffield Department of Women’s and Reproductive Health, University of Oxford, Women’s Centre, Level 3, John Radcliffe Hospital, OX3 9DU, Oxford, England.

Endometriosis – presence of endometrial-like tissue in place outside the uterus – is one of the leading causes of morbidity among reproductive-aged women and those assigned female at birth.^1^ A general unmet clinical need in endometriosis exists, driven by delayed diagnosis and limited effective treatment options, stemming from our limited understanding of the underlying mechanisms of what is recognized as a heterogeneous condition. The extent and severity of the symptoms of endometriosis, which include pelvic pain, fatigue, infertility and co-morbidities vary, considerably reducing both physical and mental quality of life and imposing an economic burden.^1^^,^^2^ Research has predominantly included selected populations in high-income countries, with limited studies from low- and middle-income countries assessing the epidemiology, health-related quality of life and economic costs of endometriosis.^3^^,^^4^ Failure to achieve greater inclusivity in endometriosis research risks further widening global disparities and hinders our ability to identify population-specific factors that influence disease presentation and severity, necessary to provide targeted diagnostic and treatment solutions.

## Health disparities

The global prevalence of endometriosis is unknown, but studies indicate that approximately 6–13% (114–247 million) of women worldwide are affected.^3^^–^^6^ Differences in the estimated prevalence of endometriosis exist, ranging from 2–11% among asymptomatic women to 5–50% among infertile women and 5–21% among women hospitalized for pelvic pain.^6^ Measuring the prevalence of endometriosis is challenging due to several factors. Reliable diagnosis often requires surgical visualization of the pelvis or access to imaging tools, due to lack of non-invasive diagnostic markers.^7^ Additionally, historical neglect in research, public health programmes and policy has contributed to general lack of awareness and the normalization of symptoms such as pain and fatigue, resulting in stigmatization.^3^^,^^8^ In some countries, access to laparoscopic investigation is focused on fertility treatment, with fertility investigations only accessible to married women.^2^ The wide heterogeneity in patient experiences, clinical presentations and disease severity further complicate the initiation of diagnostic evaluations.^1^ Access to specialized health care in low- and middle-income countries might be limited, contributing to global disparities in diagnosis and treatment.^4^^,^^5^^,^^9^

Substantial delays between first onset of symptoms and confirmed endometriosis diagnosis contribute to the overall burden. For instance, a study including 1418 women from 10 countries (Argentina, Belgium, Brazil, China, Ireland, Italy, Nigeria, Spain, the United Kingdom of Great Britain and Northern Ireland and the United States of America), who underwent their first diagnostic laparoscopic surgery for symptoms suggestive of endometriosis, showed a mean diagnostic delay of 6.7 years.^9^ Another study involving 518 women with endometriosis from the United Arab Emirates documented a mean diagnostic delay of 11.6 years, with an average of 20 years for unmarried women.^2^ A mean diagnostic delay of 6.6 years (standard deviation: 10.3 years) was reported by 1378 women with endometriosis from Spain and the World Health Organization Region of the Americas.^10^ A cross-sectional study included 410 Turkish Cypriot women with endometriosis from northern Cyprus who endured a mean time to diagnosis of 7 years (interquartile range: 15.5).^3^ The two latter studies showed that first-line hormonal treatments for endometriosis were severely underused.^3^^,^^10^ The Cyprus study highlighted the substantial economic burden for women with endometriosis, demonstrating that both medical costs and productivity losses are positively correlated with increasing severity of pain symptoms. The economic burden was particularly high in untreated populations.^3^

Research exploring health disparities in endometriosis for specific population groups is limited. While diagnostic delays seem to affect women in countries at every income level, persistent inequities in endometriosis diagnosis, treatment and care exist between populations at the subnational level. For instance, while evidence that prevalence for endometriosis differs between races and ethnicities is lacking, a systematic review from the United States revealed that, compared to white women, black women were less likely to receive an endometriosis diagnosis (odds ratio, OR: 0.49; 95% confidence interval, CI: 0.29–0.83), while Asian women were more likely to receive such diagnosis (OR:1.63; CI:95%: 1.03–2.58).^11^

Current treatment options for endometriosis primarily include surgical removal of lesions and hormonal therapies aimed at reducing tissue growth and inflammation. Data on the availability and acceptability of treatment options for women with endometriosis symptoms in low- and middle-income countries are scarce. A recent commentary^4^ highlights the limited availability of laparoscopic surgery as well as the pervasive lack of awareness about endometriosis, not only among the public but, concerningly, among health workers in the African context. Additionally, existing hormonal therapies are often viewed unfavourably due to their high costs and contraceptive effects. Large-scale studies are needed to understand the population- and country-specific disparities in health-related outcomes for those individuals with endometriosis, particularly in low- and middle-income settings.

## Research gap 

Many aspects of unmet clinical need related to endometriosis, which drive global disparities in this area, are rooted in our limited knowledge of the underlying mechanisms of this heterogeneous condition. Whether endometriosis is a single progressive disease, or a disease characterized by multiple subtypes with distinct etiologies and clinical presentations is unclear. Large-scale, inclusive research is needed to discover potential non-invasive biomarkers for diagnosis of the condition and/or potential subtypes, identify new non-hormonal treatment targets for development of therapies, and target treatments in a more precise manner based on patient characteristics and disease presentation. Precision medicine-based research aims to identify more effective prevention, diagnosis and treatment modalities by tailoring care to individuals’ genetic, environmental and social contexts. Understanding the heterogeneous nature of endometriosis is critical for developing precision medicine approaches. However, research into underlying biology, such as genetic factors and representation of non-white ancestry populations, is severely lacking. This gap restricts the global applicability of novel genetic risk scores and the effectiveness of new genetically validated treatments, further widening existing health disparities.

By using genetic and molecular data, precision medicine could enable more targeted and effective treatments for endometriosis, helping determine whether the condition consists of multiple subtypes or represents a continuum of stages. Improved diagnostics and personalized treatment plans could address unmet clinical needs, and tailoring care to specific disease stages or subtypes.

Genome-wide association studies are a primary method for the discovery of associations between heritable genetic variants and common complex conditions such as endometriosis. The International Endometriosis Genome Consortium brings together all centres generating detailed phenotypic characterization and genome-wide genotype data from women with endometriosis, and clinical or community controls from the same population. The latest consortium genome-wide association meta-analysis included about 60 000 endometriosis cases and 700 000 controls, and identified 42 genetic risk loci. Many of these loci have biologically informative roles in the pathophysiology of pain in endometriosis^12^ and provide potential drug targets for the development of novel treatments. The results also suggested differences in genetic predisposition between ovarian endometriosis compared to peritoneal and deep disease, indicating that these may be driven by distinct biological mechanisms. These differences highlight the need to explore pathways that differentiate ovarian endometriosis from other disease subtypes, which could have implications for developing subtype-specific therapeutic strategies.

These efforts have predominantly focused on white ancestry populations from Australia, European countries and the United States of America (about 98% of the study sample),^12^ similar to studies investigating molecular mechanisms through analysing the epigenome, proteome and metabolome. Genetic risk variants, potential biomarkers and treatment targets should be validated in more diverse populations. Failure to include globally representative populations in large-scale precision medicine research may result in missed genetic risk variants or novel treatment targets, while tailored drug responses and non-invasive diagnostic biomarkers may not be equally effective or sensitive across all populations, thereby perpetuating health-care inequalities. In contrast, by improving research inclusivity, we can develop better diagnostic tools and treatments, ultimately enhancing the quality of life for endometriosis patients.

## Strategies to bridge the gap

Bridging research gaps in endometriosis requires large-scale, cross-population and epidemiologically rigorous studies to identify the genetic, environmental and lifestyle factors that contribute to disease burden, and to identify novel diagnostic markers and effective treatments. The World Endometriosis Research Foundation’s Endometriosis Phenome and Biobanking Harmonisation Project provides standardized protocols for clinical data and biological sample collection from endometriosis patients, and controls to ensure comparability and replicability of results.^13^^,^^14^ Adaptation of this project’s protocols fosters consistency in research methods – enabling detailed, comparable and reliable data collection, which in turn facilitates better insights into endometriosis. These protocols are currently used by 63 institutions across 24 countries, including four lower-income and four upper-middle-income countries, and are freely accessible, facilitating collaborative research to improve disease understanding and management.

To address the lack of diversity in endometriosis research, stakeholders must advocate for greater global representation and inclusivity. Building national and international networks is crucial for enabling effective collaboration between researchers, health workers and patients. These networks can foster cooperation; strengthen research infrastructure by providing platforms for sharing technical expertise, resources and knowledge; and improve advocacy efforts for increased funding, policy changes and greater awareness of endometriosis.

As funding for endometriosis research remains an important barrier, funding bodies should prioritize this area and ensure the inclusion of underrepresented populations to better understand the disease-associated symptoms and manifestations. The recent incorporation of endometriosis as a key focus in national research policies – such as the United Kingdom’s Women’s Health Strategy and the White House Initiative on Women’s Health Research in the United States, and its recognition as an area of unmet clinical need in countries such as Australia, France and Germany represent an important step forward.

However, global initiatives and network-building across countries are necessary to prioritize endometriosis on national agendas worldwide. International collaboration between researchers that specifically advocate for inclusive precision medicine research in endometriosis are essential to bridge existing research gaps, raise awareness and improve the lives of patients worldwide.
